# Predicting students’ performance in e-learning using learning process and behaviour data

**DOI:** 10.1038/s41598-021-03867-8

**Published:** 2022-01-10

**Authors:** Feiyue Qiu, Guodao Zhang, Xin Sheng, Lei Jiang, Lijia Zhu, Qifeng Xiang, Bo Jiang, Ping-kuo Chen

**Affiliations:** 1 College of Education, Zhejiang University of Technology, Hangzhou, 310023 China; 2 College of Computer Science and Technology, Zhejiang University of Technology, Hangzhou, 310023 China; 3 Department of Educational Information Technology, East China Normal University, Shanghai, 200062 China; 4 Business School and Research Institute for Guangdong-Taiwan Business Cooperation, Shantou University, Shantou, 515000 China

**Keywords:** Computational science, Computer science, Scientific data, Statistics

## Abstract

E-learning is achieved by the deep integration of modern education and information technology, and plays an important role in promoting educational equity. With the continuous expansion of user groups and application areas, it has become increasingly important to effectively ensure the quality of e-learning. Currently, one of the methods to ensure the quality of e-learning is to use mutually independent e-learning behaviour data to build a learning performance predictor to achieve real-time supervision and feedback during the learning process. However, this method ignores the inherent correlation between e-learning behaviours. Therefore, we propose the behaviour classification-based e-learning performance (BCEP) prediction framework, which selects the features of e-learning behaviours, uses feature fusion with behaviour data according to the behaviour classification model to obtain the category feature values of each type of behaviour, and finally builds a learning performance predictor based on machine learning. In addition, because existing e-learning behaviour classification methods do not fully consider the process of learning, we also propose an online behaviour classification model based on the e-learning process called the process-behaviour classification (PBC) model. Experimental results with the Open University Learning Analytics Dataset (OULAD) show that the learning performance predictor based on the BCEP prediction framework has a good prediction effect, and the performance of the PBC model in learning performance prediction is better than traditional classification methods. We construct an e-learning performance predictor from a new perspective and provide a new solution for the quantitative evaluation of e-learning classification methods.

## Introduction

E-learning has become a typical form of education^1^ and an important part of the development of Internet-based education. Due to the impact of the COVID-19 pandemic, e-learning has been widely used worldwide due to its high temporal and spatial flexibility, low knowledge acquisition threshold, and rich learning resources. However, in this mode, teachers cannot easily perceive the learning status of their learners^2^, and questions about the quality of e-learning have been raised. The study of learning performance prediction provides a basis for teachers to adjust their teaching methods for students who may have problems by predicting students’ performance on future exams, reducing the risk of students failing to pass the course, and ensuring the quality of e-learning. Through a large number of empirical studies that investigate the relationship between e-learning behaviour and learning performance, learners’ e-learning behaviour has an important impact on learning performance. Therefore, in recent years, learning performance prediction based on learning process data has received widespread attention. The use of measurement, collection and analysis of learning process data to achieve learning performance prediction^3^ can help teachers modify teaching strategies in time and start during students’ learning processes using the role of supervision and early warning^4^.

Research notes that e-learning behaviour data are important to understanding e-learning processes. E-learning behaviour data refers to the data generated by learners in various behavioural activities performed on e-learning platforms or online teaching organizations, which can describe the activity records of learners in the learning process, specifically including the number of login platforms, the number of access to resources, the number of participants in forum discussions, the number of access to resources and other behavioural data^5^. Concurrently, e-learning behaviour involves less private information, and data collection and use are more convenient, which has an important impact on e-learning performance^6^. Therefore, researchers have conducted in-depth research on e-learning behaviour^7^ and constructed different learning performance predictors based on e-learning behaviour^8^. Learning performance prediction is usually a binary classification task that divides students into two groups of ”passed” or ”failed” to predict the possibility of passing the test in the future^9^. Because predictive learning is the primary advantage of machine learning technology, it is often used to train the learning performance prediction model using a simple method^10,11^. Although this type of predictor can achieve good prediction results, it has certain limitations. First, regarding low generalizability and high computational complexity, a large number of e-learning behaviour data of different dimensions are captured and recorded during the e-learning process. Constructing an e-learning performance predictor is prone to overfitting and high computational complexity. Feature selection can be used to retain key learning behaviours to reduce model operational costs, which is of practical significance for online platforms to provide high-accuracy and low-time-consuming learning performance prediction services. Second, the single input method of e-learning behaviour data requires that e-learning predictors generally use e-learning behaviour data directly as input variables. Few predictors will consider the combined effect of learning behaviour data (i.e., perform feature fusion processing) on the same type of learning behaviour data and then use it for training. Last, key learning behaviour indicators are not standardized, and those found by different researchers ares different. This field of study has failed to identify key behaviour indicators that can be used to effectively predict learning performance^12,13^; thus, the results of the prediction models are frequently affected by platform-specific learning behaviours, which affects the model’s mobility.

To solve these problems, we propose the behaviour classification-based E-learning performance prediction framework (BCEP prediction framework), summarize the classic e-learning classification methods, analyse the e-learning process in detail, propose the process-behaviour classification model (PBC model), and construct an e-learning performance predictor based on the PBC model. The primary contributions of this article are as follows. First, the BCEP prediction framework proposed in this paper includes four steps: data cleaning, behaviour classification, feature fusion, and model training. Compared with general learning performance predictors, this framework yields more accurate predictions of student achievement. During training, computational complexity is reduced, and the model’s mobility and versatility are increased in the application process. Second, the PBC model proposed in this paper divides e-learning behaviours into four categories. The learning performance predictor based on the PBC model is shown to perform markedly better than existing typical classification methods .

This article is composed of 7 summaries, and the remainder of its content is organized as follows. Section 2 summarizes the development status of e-learning performance prediction, focusing on the prediction indicators and methods of e-learning performance prediction. Section 3 describes the BCEP prediction framework in detail. Section 4 reviews the existing learning classification models and designs a new e-learning behaviour classification method-PBC model. Section 5 describes the experiments used to verify the effectiveness of the BCEP prediction framework and PBC model. In Section 6, experimental results are systematically analysed and discussed. Last, Section 7 provides conclusions and prospects for future research.

## Related work

### Prediction indicators of e-learning performance

E-learning performance predictors are generally summarized as tendency indicators and behavioural performance indicators^14^. Tendency indicators are inherent attributes of themselves; primarily static data, which are generally collected before the start of the course or semester, such as socioeconomic status^15^, historical academic records^16^ and gender^17^, are common indicators of propensity. Many researchers have used propensity indicators to develop learning early-warning models to predict students’ learning in a course, a semester, and other stages. Although the predictors established by these studies achieve good performance, they ignored the role of learning behaviour records. For example, many studies used students’ historical performance or demographic data that were not related to learning. Although these studies can predict learning performance through learner characteristics, this method ignored that most of the tendency indicators were not in the student’s and the teacher’s control, and the students’ changes in curriculum were ignored^18^. In addition, there is a privacy problem with preference indicators, and personal data collected by educational institutions cannot be shared publicly. The behavioural performance index (i.e., the dynamic index reflected by the learner in the learning process^19–21^) generally did not have such a problem. E-learning behaviour data can accurately describe the time and energy that students spend on a specific course, such as the frequency of access to course materials^22^ and the frequency of online discussions^23^. Some studies also tried to use the combination of two indicators to complete learning prediction^24^ but encountered problems related to increasing computational costs.

The accumulation of educational big data and the emergence of new methods of connecting and exchanging information have laid the foundation for e-learning behaviour research. Learners’ learning behaviour data are important when analysing changes in learners’ behaviour, preferences, and ability levels^25^, which promotes related research on learning performance prediction based on learning behaviour. Learning input theory explains the relationship between learning behaviour and learning performance^26^ and states that learning behaviour is a key factor affecting learning performance and an important indicator for predicting learning performance^27^. Concurrently, many studies have confirmed that there is a significant correlation between student online activities and academic performance^28,29^, and observing learning activities at a finer-grained level can strengthen the grasp of learning conditions and promote constructive learning^30^. Therefore, many researchers have explored the correlation between e-learning behaviour and learning performance, and used e-learning behaviour to predict learning performance. For example, Qi^31^reported that there is a significant positive correlation between learners’ e-learning behaviour and the learning effect. Liang et al.^32^ recorded student data through a curriculum management system and used regression analysis to find a correlation between learning experience, learning behaviour and learning performance. Comer et al.^33^ found that in the e-learning environment, collaborative communication behaviour will deepen students’ understanding of knowledge and encourage students to achieve certain learning achievements. Kokoç and Altun^34^ used learning interaction data to predict the learning performance of online learners and found that the access behaviour of learning content, books, forums, and course activities can significantly affect learning outcomes. Some studies looked for a relationship between a certain behavioural activity or several behavioural activities and learning performance. Zheng et al.^35^ found that there is a positive correlation between the number of logins and the final grades of students. Qureshi et al.^36^ used a questionnaire survey method to find that cooperative learning and learning participation play an intermediary role between social factors and learning performance, and verified that collaborative learning behaviours promote learning performance in e-learning. Shen^37^ noted that the proportion of learners’ homework completion and video completion rate in e-learning affect learning.

Based on the literature about learning performance prediction using learning behaviours, analyses of e-learning behaviour is frequently limited to independent e-learning behaviours. Few studies have explored the internal associations and differences between e-learning, specifically categorizing and analysing e-learning behaviours. In previous studies that used learning behaviour classification as primary predictor indicators, researchers only used independent e-learning behaviour data as the input of predictor training instead of the fusion data of learning behaviour classification, which reduced the importance of learning behaviour classification.

### Prediction algorithm of e-learning performance

In e-learning performance prediction research, the selection of predictive indicators occupies an important position, and prediction methods also play a key role, particularly feature selection and algorithm selection, which can markedly affect the prediction effect. Therefore, it is necessary to identify relevant research and applications of machine learning and feature selection.

An increasing number of studies have confirmed that when constructing predictive models, multiple data points cannot always guarantee a higher predictive ability. Unnecessary features will affect the generalizability of the model and increase the computational cost of the model. For example, Akram et al.^38^ used ten prediction algorithms to predict students with learning difficulties through assignment submission behaviour and found that the prediction performance of all algorithms decreased as the number of input data increased. It is thus necessary to select behavioural features that are meaningful for learning performance from the sample data and then input them into the model for training and learning; thus, feature selection is necessary^39^. Three methods can be used for feature selection: the filter method, the wrapper method, and the embedded method. Madichetty et al.^40^ verified that the selection of key features is helpful for classification prediction. The filtering feature selection method has the advantages of strong independence, fast running speed and low computational complexity in machine learning algorithms, but makes it difficult to completely delete redundant features when there are many redundant features and high target relevance^41^. Wrapping methods can be independent of machine learning models but typically have high computational costs^42^. The embedded method embeds feature selection into other algorithms and selects new features during the training process, which can effectively improve the efficiency of model learning^43^.

In addition, machine learning algorithms have unique advantages in solving classification problems. For example, Huang and Lin et al.^44^ proposed a multimodal information perception method for flexible manipulators based on machine learning methods to complete gestures, object shapes, sizes and weights to recognize tasks, compared the recognition accuracy of optical sensor information (OSI), pressure sensor information (PSI) and dual sensor information (DSI). They found that the KNN algorithm with DSI performed better than other with regard to recognition accuracy. Cao and Zhang et al.^45^ used and improved the deep learning method; used the multitask cascaded convolution network (MTCNN) method to locate the face of cartoon characters; performed face detection and face feature point detection; and recognized the image emotion of cartoon style. Muhammad and Liu et al.^46^ extended the application of machine learning to the field of language recognition and translation by sharing dictionary embeddings between the parent language and the child language without using reverse translation or manual noise injection and proposed a language-independent hybrid transfer learning (HTL) method to solve the problem of data sparseness in low-resource languages (LRLs). Machine learning technology has gradually emerged in the development of the learning analysis process, which facilitates the collection and analysis of student and environmental data^47^. In recent years, many machine learning classification algorithms have been applied to the field of learning performance prediction. For example, Jiang et al.^48^ built a predictor based on logistic regression, which combined students’ first week of homework performance and social interaction behaviour to predict learners’ performance in the course. Aziz et al.^49^ selected five parameters, race, gender, family income, college enrolment mode and average grade point, and used the naïve Bayes classifier to predict the average grade point. Ahuja and Kankane^50^ used the K-nearest neighbour algorithm to predict the results of students’ academic acquisition based on the previous academic performance and non-academic factors of college students. Asif et al.^51^ used the decision tree algorithm to predict students’ performance at the end of the four-year study plan. Jie-ping et al.^52^ proposed a performance prediction method that combined fuzzy clustering and support vector machine regression based on students’ historical performance and behavioural habits.

## Behaviour-based classification of the e-learning performance prediction framework

Researchers typically use the behaviour of each e-learning category as an independent predictor of the performance of e-learning to build predictive models. However, different e-learning behaviours have potential correlations and can be classified into different behaviour categories according to different rules. This research innovatively constructs a learning performance predictor from the perspective of behaviour categories and proposes the behaviour classification-based E-learning performance prediction framework (BCEP prediction framework).

**Figure 1:**
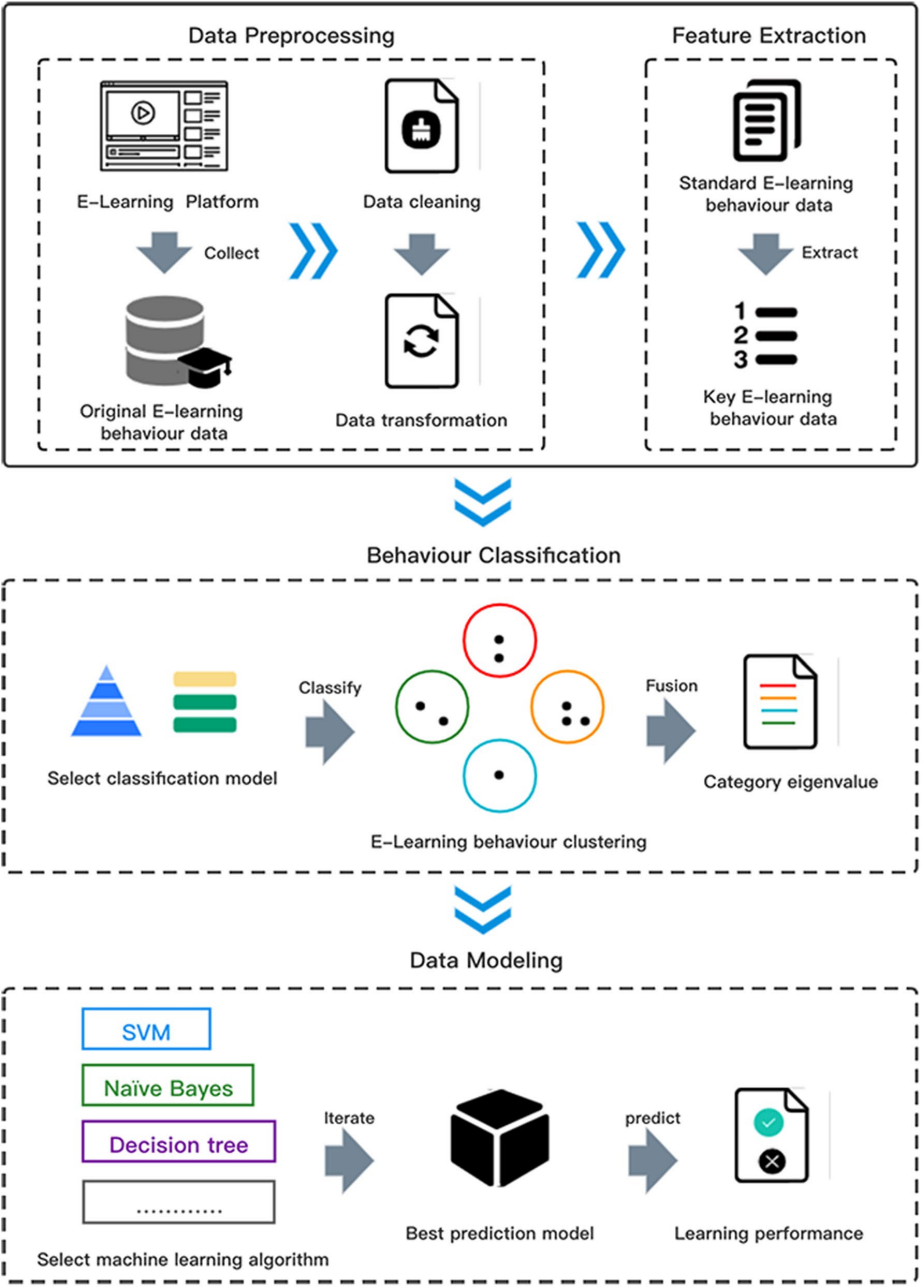
Behavior-based classification of e-learning performance prediction framework.

The BCEP prediction framework describes the complete process of implementing learning performance predictors through e-learning behaviour categories, as shown in Fig. 1. The prediction framework includes four core links: (1) data pre-processing, which includes data cleaning and conversion from the original e-learning behaviour data obtained by the e-learning platform obtain standardized e-learning behaviour data; (2) feature selection, which is performed on pre-processed e-learning behaviour data to obtain key e-learning behaviours; (3) feature fusion, which classifies core learning behaviours according to specific rules, constructs a collection of behaviour categories, and then performs feature fusion to obtain the category feature value of each type of e-learning behaviour; and (4) model training, which builds an e-learning performance predictor based on a variety of machine learning algorithms.

(1) Data pre-processing

The quality of e-learning behaviour data directly affects the accuracy of predictive models. Therefore, the first step is to clean the e-learning behaviour data obtained from the e-learning platform. There is no unified process for data cleaning, but the method should be selected according to the real situation of the data to manage missing values, duplicate values, and abnormal values. Concurrently, e-learning behaviours recorded by e-learning platforms are often not of a single dimension, e-learning behaviour data of different dimensions are often not numerically comparable, and feature selection cannot be performed. The proposed framework solves this problem by standardizing e-learning behaviour data in different dimensions with Z scores.

We define the original e-learning behaviour set $B \left\{ b_{1}, b_{2}, \ldots . ., b_{n}\right\}$ and the standard e-learning behaviour set $B\left\{ b^{\prime }, b_{2}^{\prime }, \ldots , b_{n}^{\prime }\right\}$ . Where $b_{n}$ is the n-th e-learning behaviour recorded by the e-learning platform, and $b_{n}^{\prime }$ is the n-th e-learning behaviour after standardization. Concurrently, the original e-learning behaviour data and the standard e-learning behaviour data are defined, where n is the n-th e-learning behaviour, and m is the m-th data of the current e-learning behaviour. For example, $d_{nm}$ is the second behaviour data of the first type of e-learning behaviour recorded by the e-learning platform, and the formula for $d_{nm}^{\prime }$ is as follows:

1 $$\begin{aligned} \mathrm {d}_{\mathrm {nm}}^{\prime }=\frac{\mathrm {d}_{\mathrm {nm}}-\mu _{\mathrm {b}_{\mathrm {m}}}}{\sigma _{\mathrm {b}_{\mathrm {m}}}} \end{aligned}$$

Where $\mu _{\mathrm {b}_{\mathrm {m}}}$ is the average value of the n-th type of e-learning behaviour data, and $\sigma _{\mathrm {b}_{\mathrm {m}}}$ is the variance of the n-th type of e-learning behaviour data.

(2) Feature selection

Feature selection can select relevant features that are beneficial to the training model from all features, thereby reducing the feature dimension and improving the generalizability, operating efficiency and interpretability of the model. This framework uses the variance filtering method to perform feature selection on standardized e-learning behaviour data. The variance filtering method uses the variance of each feature itself to filter the features. The smaller the variance of the feature, the lower the difference of the sample on this feature, and the smaller the distinguishing effect of the feature on the sample. The threshold is an important parameter of the variance filtering method, which represents the threshold of variance; thus, features with variance less than the threshold will be discarded.

We define the characteristic value set of e-learning behaviour $V \left\{ v_{1}, v_{2}, \ldots , v_{n}\right\}$, where $v_{n}$ is the characteristic value of the n-th e-learning behaviour, and its formula is as follows:

2 $$\begin{aligned} V_{n}=\frac{\sum _{i=1}^{m}\left( d_{n i}^{\prime }-\mu _{b^{\prime }_{m}}\right) ^{2}}{n} \end{aligned}$$

where $\mu _{b^{\prime }_{m}}$ represents the average value of the n-th standard e-learning behaviour data. The elements in traversal V are compared with the variance threshold. If the current e-learning behaviour feature value is greater than the threshold, the corresponding e-learning behaviour is added to the key e-learning behaviour set; otherwise, it is not added.

(3) Feature fusion

First, according to the e-learning behaviour classification model, the key e-learning behaviour is divided into different e-learning behaviour clusters. We assume that the classification Model M is composed of n types of e-learning behaviour categories ( ie.,$M\left\{ C_{1}, C_{2}, \ldots , C_{n}\right\}$). After dividing the e-learning behaviour categories, n e-learning behaviour clusters are generated, and each type of e-learning behaviour cluster includes a varying number of e-learning behaviours, such as $C_{1}\left\{ b_{1}, b_{2}, \ldots , b_{n}\right\}$ , where, $b_{n}$ is the n-th e-learning behaviour that meets the standard of $C_{1}$ .

Then, feature fusion is performed on each e-learning behaviour cluster to obtain the corresponding feature value of the e-learning behaviour category $C_{1}\left\{ b_{1}, b_{2}, \ldots , b_{n}\right\}$ as an example. The calculation formula of its category feature value is as follows:

3 $$\begin{aligned} V_{c_{i}}=\lambda \cdot \max \left\{ V_{b_{1}}, V_{b_{2}} \ldots , V_{b_{i}}\right\} +(1-\lambda ) \cdot \frac{\sum _{i=1}^{n} V_{b_{i}}}{n}, \lambda =(0, 1) \end{aligned}$$

In Eq.3, $V_{b_{i}}$ is the $b_{i}$ characteristic value of the behaviour, $\lambda =0$ means the student has passed the curriculum, $\lambda =1$ means failed. Similarly,we construct the feature value set of e-learning behaviour category $V_{c}\left\{ V_{c1}, V_{c2}, \ldots , V_{ci}\right\}$ where $V_{C_{i}}$ is the category feature value of the $C_{1}$ e-learning behavior.

(4) Model training

In the model training session, classic machine learning methods such as SVC, NaÃve Bayes, KNN and Softmax are selected, and the e-learning behaviour category feature value set $V_{C}$ is used as the feature data to train the e-learning performance prediction model. After many iterations, the best e-learning performance prediction model is selected to predict the e-learning performance of e-learners.

## E-learning process—behaviour classification model

The e-learning behaviour classification model is an important component of the BCEP prediction framework that directly affects the prediction effect of the e-learning performance prediction model. This paper summarizes the current mainstream e-learning behaviour classification methods, as shown in Table 1.

**Table 1:** Classification and basis of e-learning behaviour.

Paper	E-learning behavior categories	Classification basis	Number of categories
Moore^53^	Learner–content interaction, learner–instructor interaction, learner–learner interaction	Learning interactive object	3
Hillman et al.^54^	Learner–content interaction, learner–instructor interaction, learner–learner interaction, learner–interface interaction	Learning interactive object	4
Hirumi^55^	Learner–self interactions, learner–human and non-human interactions, learner–instructor interactions	Learning interactive object	3
Peng et al.^56^	Information retrieval learning behavior, information processing learning behavior, information publishing learning behavior, interpersonal communication behavior, problem-solving learning behavior	learning behavioral diversity	5
Malikowski et al.^57^	Most used category, moderately used categories, rarely used categories	Feature adoption rate of typical VLE	3
Veletsianos et al.^58^	Activities that are digital, activities that are not digital, activities that are social, activities that are individual	MOOCs course features	4
Lijing Wu et al.^59^	Independent learning behavior, system interaction behavior, resource interaction behavior, social interaction behavior	Basic elements of e-Learning space	4
Fti Wu et al.^60^	Student–student interaction, student–teacher interaction, student–content interaction, student–system interaction	Learning behavioral diversity	4

**Figure 2:**
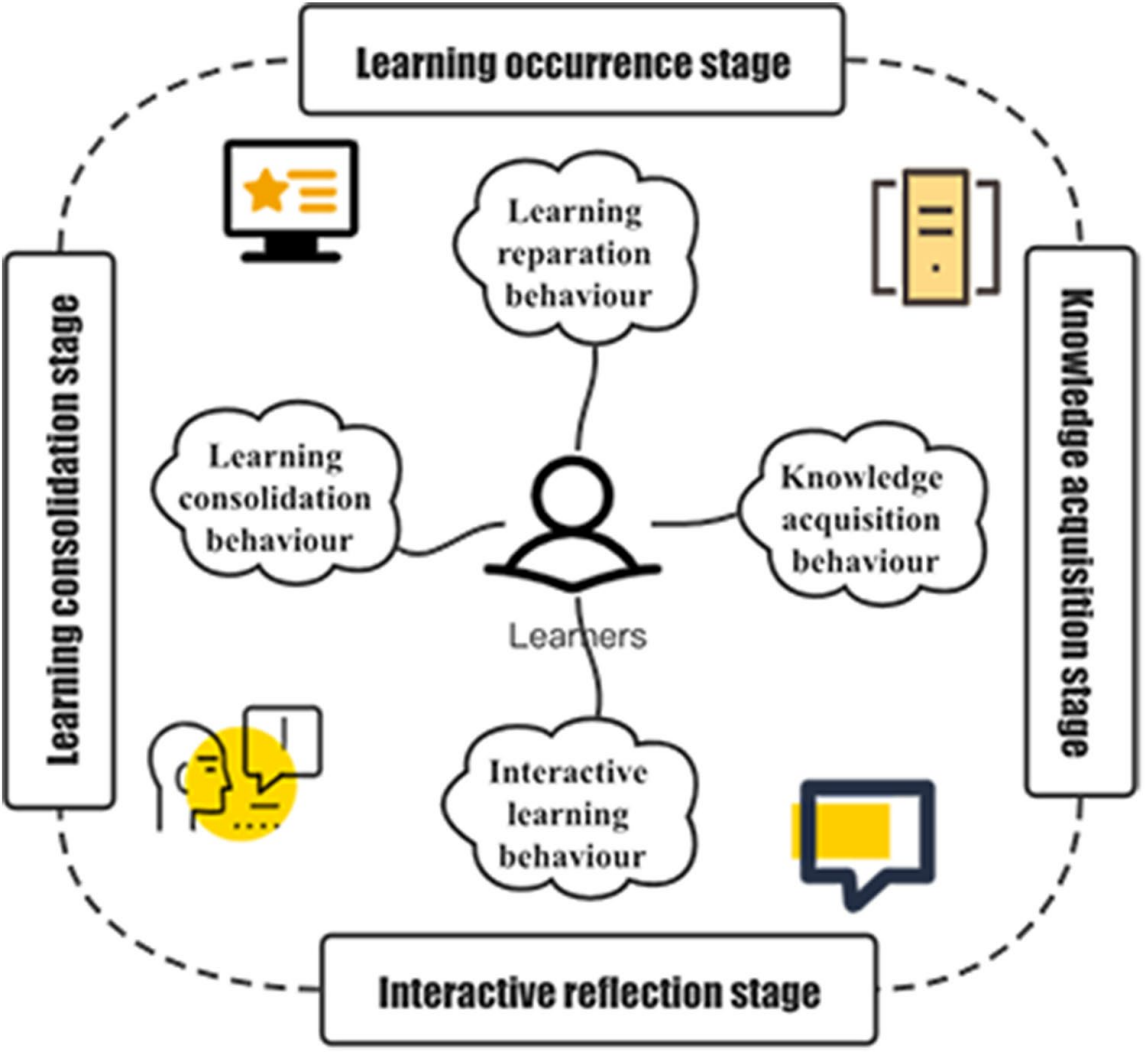
The process-behaviour classification model (PBCM.

Table 1 shows that most researchers use interactive objects as the basis for the classification of learning behaviours. The primary interactive objects include learning systems, resource content, learning communities, and learners themselves. However, when learners are in different stages of learning, they often engage in different learning behaviours, but the interactive objects may be the same. The classification method based on interactive objects does not fully consider the process of learning. Based on these ideas, this study constructed an e-learning behaviour classification model based on the e-learning process-process-behaviour classification model (PBC model), as shown in Fig. 2 .

The e-learning process primarily includes the learning stage, the knowledge acquisition stage, the interactive reflection stage and the learning consolidation stage^5^. The learning stage is the preparation process for learners to officially start e-learning; the knowledge acquisition stage is the most important e-learning process and is also the process by which learners initially acquire knowledge; the interactive reflection stage is a process in which learners interact with teachers and peers and reflect on themselves during the interaction; and the stage of learning consolidation is the process by which learners consolidate internalized knowledge. The model is centred on online learners, and according to the e-learning process, learning behaviour is divided into learning preparation behaviour (LPB), knowledge acquisition behaviour (KAB), interactive learning behaviour (ILB), and learning consolidation behaviour (LCB).

1. Learning preparation behaviour (LPB ) occurs during the learning stage and is the most basic behaviour of learners in e-learning. Specifically, LPB includes behaviours such as logging in to the learning platform, accessing the primary page of the course, and accessing the course activity interface.
2. Knowledge acquisition behaviour (KAB) occurs during the knowledge acquisition stage and is the behaviour of online learners directly acquiring knowledge. KAB primarily includes activities such as browsing course content resources, participating in course activities, watching course videos, and accessing resource links.
3. Interactive learning behaviour (ILB) occurs in the interactive reflection stage and is one of the key learning behaviours in e-learning. ILB has been proven to have a positive effect on the continuity and learning effect of e-learning^61^. Its specific manifestations include participating in seminars, publishing forums, replying to forums, asking questions to teachers, etc..
4. Learning consolidation behaviour (LCB) occurs in the stage of learning consolidation and refers to the behaviour of learners to strengthen the degree of knowledge mastery, primarily including proposing postclass reflections and completing postclass tests.

## Experimental design

Experiments are used to compare the prediction performance of the predictor in the traditional framework and the BCEP prediction framework based on the PBC model proposed in this study to verify the effectiveness of the proposed framework. Its predictors include six machine learning methods: SVC (R), SVC (L), Naïve Bayes, KNN (U), KNN (D) and softmax^10^. We selected the accuracy rate, F1-score and Kappa coefficient as the quantitative indicators to evaluate the prediction performance. To fully verify the BCEP prediction framework, the evaluation indicators also include the time required for the experiment to complete the prediction.

We used a XiaoXin AirPlus14 Laptop to build the experimental environment, which consists of an AMD Ryzen 5600u processor, NVIDIA GeForce MX450 graphics card and a 500-GB hard disk. In terms of software, programming was performed in the Jupyter lab programming platform of the Windows 10 operating system and in the Python programming language for experiments.

### Data sources

The Open University Learning Analytics Dataset (OULAD)^62^ is considered to be one of the most comprehensive international open datasets in terms of e-learning data diversity, including student demographic data and interaction data between students and VLE. The role of Open University in developing this dataset is to support research in the field of learning analysis by collecting and analysing learner data to provide personalized guidance and optimize learning resources. The dataset contains 7 course modules (AAA   GGG), 22 courses, e-learning behaviour data and learning performance data of 32,593 students. The collection phase of the entire dataset includes collection, selection, and anonymization. We use SAS technology to create a data warehouse to collect data, select data containing student information from 2013 to 2014, and finally anonymize the data. Its design types are time series design, data integration objective and observation design; its measurement type is learning behaviour; its technology type is digital curation, and its factor type is temporal_interval. In this experiment, the DDD course module with the most sample data was selected, and the e-learning data of 6,272 learners who participated in the DDD course were used as the data source for training and verifying the e-learning performance predictor. When learning DDD courses, learners performed 12 e-learning behaviours, as shown in Table  2.

**Table 2:** E-learning behavior and coding of DDD courses.

number	E-learning behavior	Explanation	Coding
1	Homepage	Access the main interface of the learning platform	H
2	Page	Access the course interface	P
3	Subpage	Access the course sub-interface	S
4	Glossary	Access the glossary	G
5	Ouwiki	Query with Wikipedia	W
6	Resource	Search platform resources	R
7	URL	Access course URL link	U
8	Oucontent	Download platform resources	T
9	Forumng	Participate in the course topic forum	F
10	Oucollaborate	Participate in collaborative exchange activities	C
11	Ouelluminate	Participate in simulation course seminars	E
12	Externalquiz	Complete extracurricular quizzes	Q

### Experimental design for validation of the BCPF prediction framework

(1) Experimental program

This experiment sets up three experimental groups to compare and verify the effectiveness of the BCEP prediction framework. The difference between the three experimental groups lies in the feature data used to train the learning performance predictor. Group 1 uses online behaviour data that have only undergone data pre-processing as characteristic data. Group 2 uses data that have undergone feature selection but not feature fusion as characteristic data. Group 3 follows the BCEP prediction framework and uses existing features. The data that have undergone feature fusion are selected as feature data. In this experiment, 6 machine learning methods were selected, 18 learning performance predictors were constructed based on the 3-feature data above , and the effectiveness of the BCEP prediction framework was verified by comprehensively comparing the prediction results of the 18 learning performance predictors.

(2) Feature selection

The experimental group using feature selection uses the variance filtering method to feature a selection of 12 online learning behaviours in the dataset, and 8 of them are selected as the feature data for constructing the learning performance predictor according to the variance threshold. The feature data of the three experimental groups after feature selection are shown in Table 3:

**Table 3:** Feature indication.

Online learning behavior coding	Group 1	Group 2	Group 3
H	$\checkmark$	$\checkmark$	$\checkmark$
P	$\checkmark$	$\checkmark$	$\checkmark$
S	$\checkmark$	x	x
G	$\checkmark$	x	x
W	$\checkmark$	x	x
R	$\checkmark$	$\checkmark$	$\checkmark$
U	$\checkmark$	$\checkmark$	$\checkmark$
T	$\checkmark$	$\checkmark$	$\checkmark$
F	$\checkmark$	$\checkmark$	$\checkmark$
C	$\checkmark$	$\checkmark$	$\checkmark$
E	$\checkmark$	x	x
Q	$\checkmark$	$\checkmark$	$\checkmark$

(3) Feature fusion

**Figure 3:**
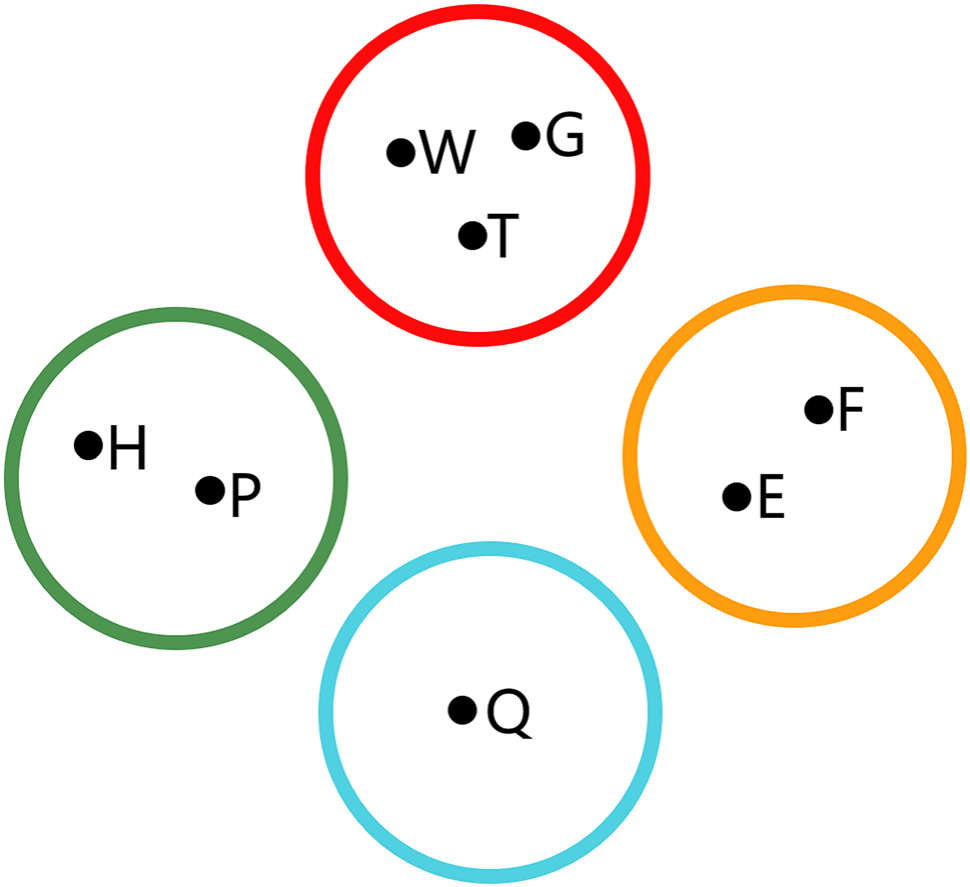
Schematic diagram of e-learning behaviour classification in Group 3.

Before using feature fusion, the experimental group classifies e-learning behaviours through the PBC model and then performs feature fusion on behaviour clusters to obtain behaviour category feature values. Finally, the behaviour category feature value is used as the feature data for building the learning performance predictor. A schematic diagram of the learning behaviour classification of Group 3 is shown in Fig. 3:

In this experiment, the number and dimensions of data after feature fusion in the 3 experimental groups are shown in Table 4.

**Table 4:** Experimental group data and dimensions.

Group	Data	Number of data	Dimension
Group 1	H,P,S,G,W,R,U,T,F,C,E,Q	12	12
Group 2	H,P,,G,W,,,T,F,E,Q	8	8
Group 3	H,P,,G,W,,,T,F,E,Q	8	4

### Experimental design for the validation of the PBC model

(1) Experimental program

The classification of e-learning behaviours are generally limited to theoretical research, and its role and scientific validity in learning performance prediction are difficult to verify. This experiment follows the learning performance prediction framework based on behaviour classification and selects three representative behaviour classification models for comparison with the PBC model, including Moore^53^ e-learning behaviour classification (a total of three types of behaviour), Wu^60^ e-learning behaviour classification (a total of four types of behaviours) and Peng^56^ e-learning behaviour classification (a total of five types of behaviours). We use 6 classic machine learning methods to construct 24 learning performance predictors and verify the effectiveness of the PBC model by comprehensively comparing the prediction results of 24 learning performance predictors.

(2) Behaviour classification

**Figure 4:**
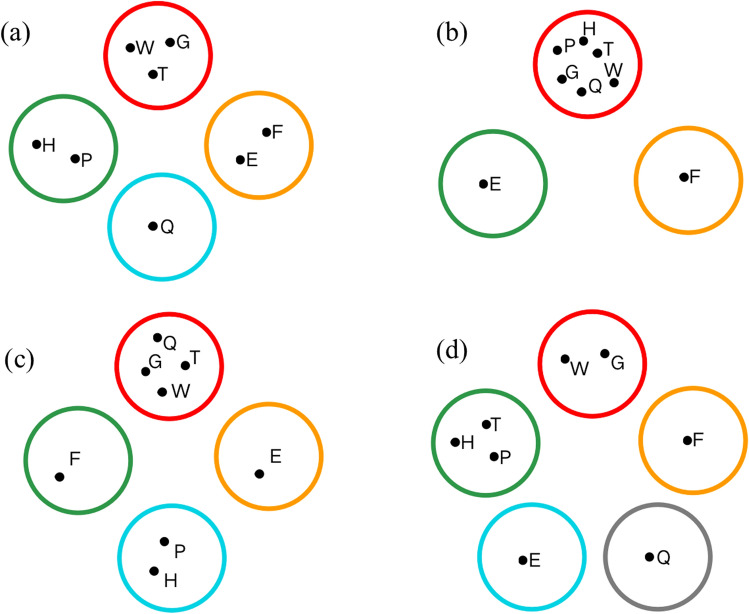
Online student behaviour classification: (**a**) PBCM, (**b**) Moore, (**c**) Wu, and (**d**) Peng.

First, feature selection is performed on 12 e-learning behaviours in the original dataset, and 8 e-learning behaviours are selected according to the variance threshold. Then, the 8 e-learning behaviours are classified according to the classification methods of the 4 experimental groups, and the classification results are shown in Fig. 4:

### Predictor implementation and evaluation

This experiment selects six machine learning algorithms that are currently widely used in the field of learning and prediction, such as SVC (R), SVC (L), Naïve Bayes, KNN (U), KNN (D) and softmax. We divided the original data $70:30\%$ for training and testing^63^, and the expected output value of the predictor was ”qualified” or ”unqualified”.

Common indicators that are used to evaluate predictors include accuracy (ACC), F1-score (F1), and Kappa coefficient (K). ACC is considered to be the most commonly used measurement index, which refers to the proportion of the number of correctly classified samples to the total number of samples, and its formula is as follows:

4 $$\begin{aligned} \mathrm {ACC}=\frac{\sum \mathrm {T}_{\mathrm {p}}+\sum \mathrm {T}_{\mathrm {n}}}{\sum \mathrm {totaldata}} \end{aligned}$$

where $T_{p}$ is the number of positive samples correctly predicted, $T_{n}$ is the number of negative samples correctly predicted, and *totaldata* is the total number of samples.

However, because the accuracy rate cannot fully evaluate the prediction model, further analysis of the F1-score (F1) and Kappa coefficients is required. The formula of *F*1 is as follows:

5 $$\begin{aligned} F_{1}=2 \frac{P \times R}{P+R} \end{aligned}$$

The formula of Kappa is as follows:

6 $$\begin{aligned} K=\frac{p_{o}-p_{e}}{1-p_{e}}=1-\frac{1-p_{o}}{1-p_{e}} \end{aligned}$$

where$P_{o}$ is the observed coincidence ratio, and $P_{e}$ is the coincidence ratio due to randomness.

## Result analysis and discussion

This section presents the experimental results, including ACC, F1, Kappa, and the prediction time of each experimental group as calculated by the six machine learning methods. The BCEP prediction framework and the PBC model are verified by analysing these data.

### BCEP prediction framework validation

**Figure 5:**
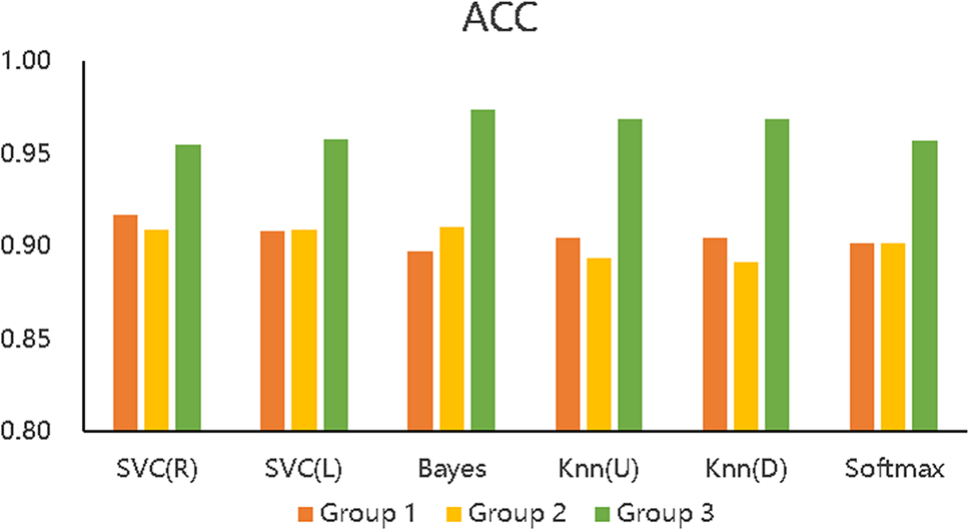
Accuracy of the three types of prediction models.

**Figure 6:**
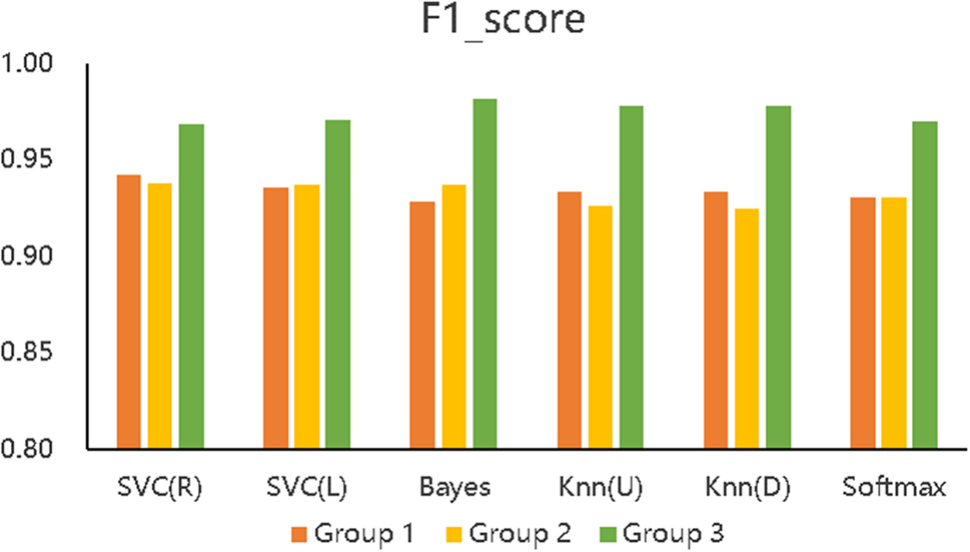
F1-score of the three types of prediction models.

**Figure 7:**
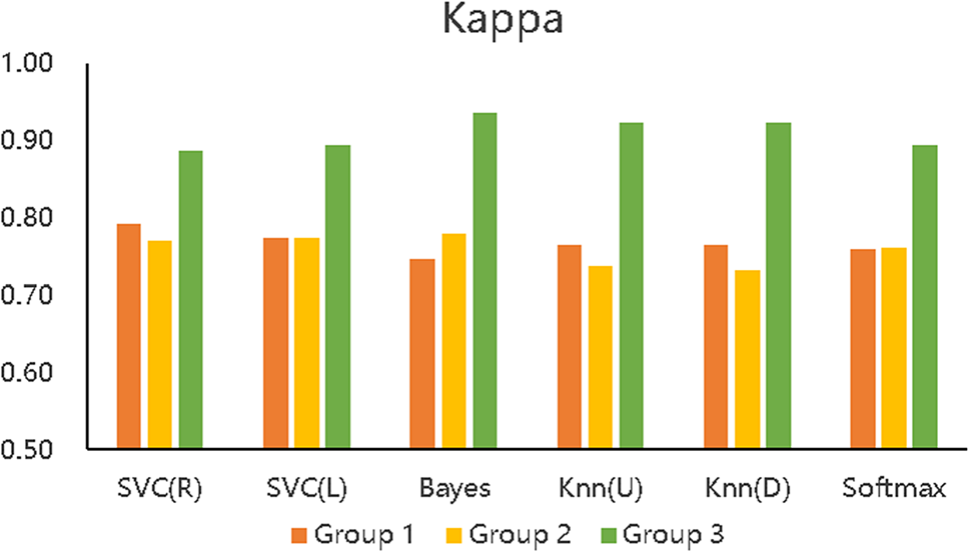
Kappa of the three types of prediction models.

**Figure 8:**
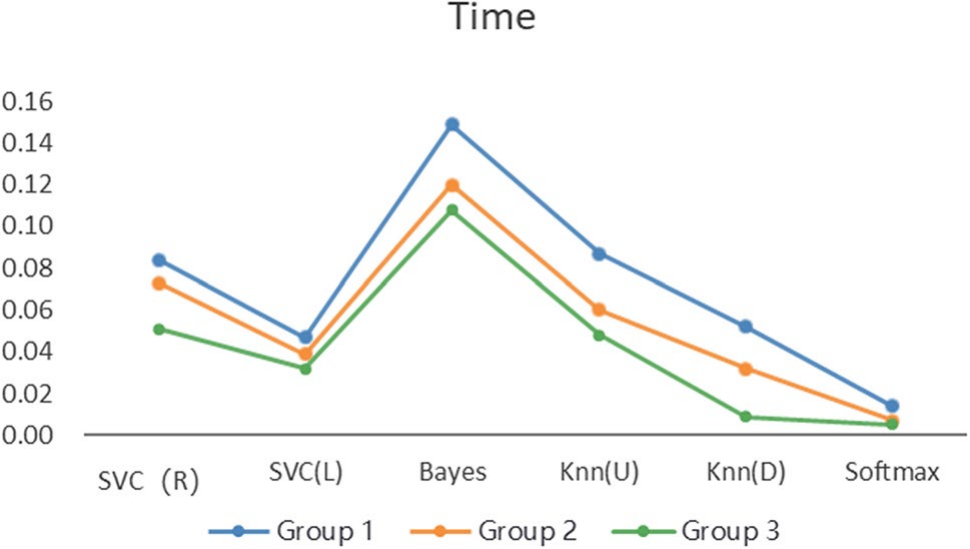
Computation time required for each of the three types of prediction models.

We designed 3 control groups (different feature data) and used 6 common machine learning algorithms to build 3 types (18) of learning performance predictors to discuss the effectiveness of the BCEP prediction framework based on the prediction effects of the predictors. The ACC, F1, and Kappa of the three types of learning performance predictors are shown in Figs. 5, 6, and 7 , respectively. To verify the role of feature selection, we also compared the experimental time of different experimental groups to complete the prediction task, as shown in Fig. 8.

Figure 5 describes the accuracy of the 6 algorithms (SVC(R), SVC(L), naïve Bayes, KNN(U), KNN(D), and softmax) with three different data processing methods. The accuracy rate of Group 1 is distributed between 89.7% and 91.65%, that of Group 2 is between 89.15% and 91.00%, and that of Group 3 is between 95.44% and 97.40%. The accuracy of Group 3 is also shown to be higher than that of the other two experimental groups with all six algorithms, which means that the prediction accuracy based on the proposed BCEP prediction framework is the highest. Experimental results show that the F1-score of Group 1 is between 0.9280$\sim$0.9423, that of Group 2 is between 0.9246$\sim$0.9374, and that of Group 3 is between 0.9685$\sim$0.9818; thus, Group 3 has the highest F1-score. The Kappa of Group 1 is between 0.7473$\sim$0.7916, that of Group 2 is between 0.7310$\sim$0.7820, and that of Group 3 is between 0.8865$\sim$0.9364; thus, Group 3 achieve markedly higher Kappa values. Lastly, the computational time required for Group 1 under each algorithm is 0.0139 s$\sim$0.1489 s, that for Group 2 is 0.0070 s$\sim$0.1199 s, and that for Group 3 is 0.0050 s$\sim$0.1080 s; thus, the computational time required for Group 2 is less than that of Group 1, and Group 3 is the fastest in obtaining prediction results in each algorithm.

In addition, we can compare the indicators of Groups 1, 2, and 3 using Figs. 5, 6, 7, and 8, although the prediction performance of Groups 1 and 2 on different algorithms has both advantages and disadvantages. In general, after applying the feature selection strategy, Group 2 reduces the feature dimension from 12 to 8, the prediction effect is still near that of Group 1, and the speed is increases by 23.56%. These results show that the feature selection strategy can reduce the predictor’s training parameters while maintaining the predictor’s predictive performance and can reduce the time complexity of the operation. Group 3 is based on Group 2, according to the idea of behaviour classification and adopts a feature fusion strategy to further reduce the feature dimension from 8 to 4, and all indicators for each of the 6 machine learning algorithms are better than those of Group 1 and Group 2. Further analysis of Figs. 5, 6, and 7 shows that the accuracy increased by 5.8% and 6.1% on average compared to Groups 1 and 2; the F1-score increased by 4.06% and 4.24%, respectively; Kappa markedly increased by 14.24% and 15.03%, respectively; and the computation time decreased by 41.57% and 23.56%, respectively. The learning performance prediction framework based on behaviour classification proposed in this paper is thus effective in real scenarios. Building a learning performance predictor with this framework can reduce the dimensionality of feature data and also markedly improve prediction performance compared to traditional methods that only use data pre-processing or feature selection strategies to build learning performance predictors based on pre-processing.

### PBC model validity verification

**Figure 9:**
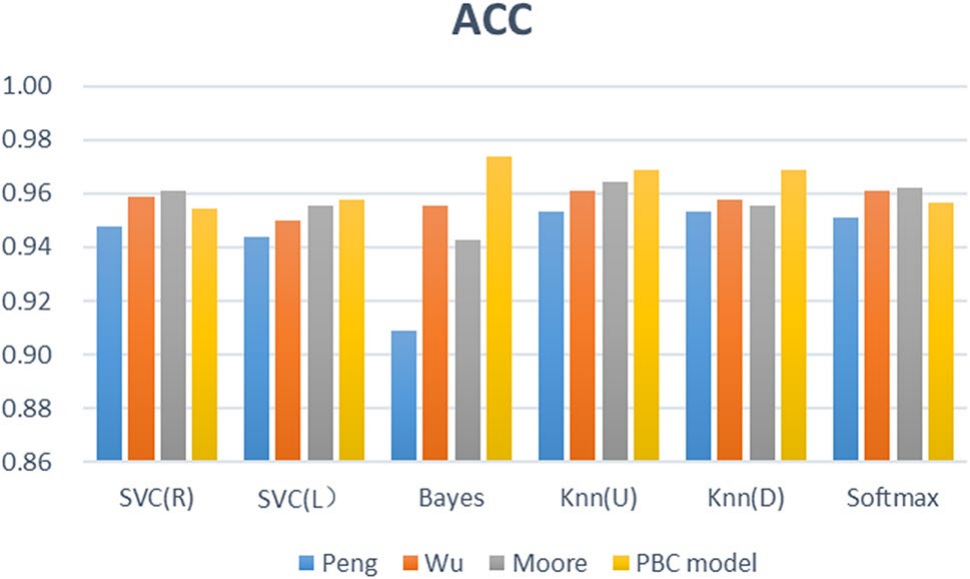
Accuracy of the four types of prediction models.

**Figure 10:**
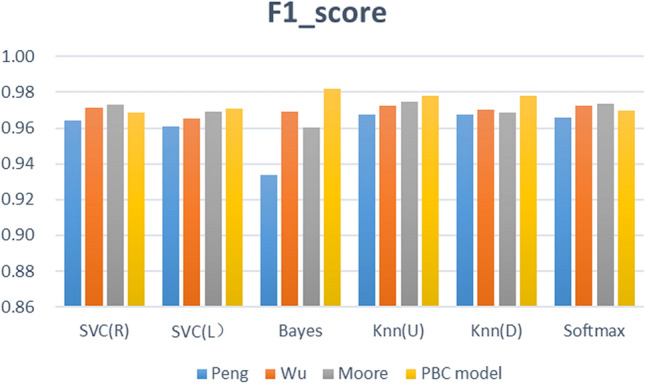
F1-score of the four types of prediction models.

**Figure 11:**
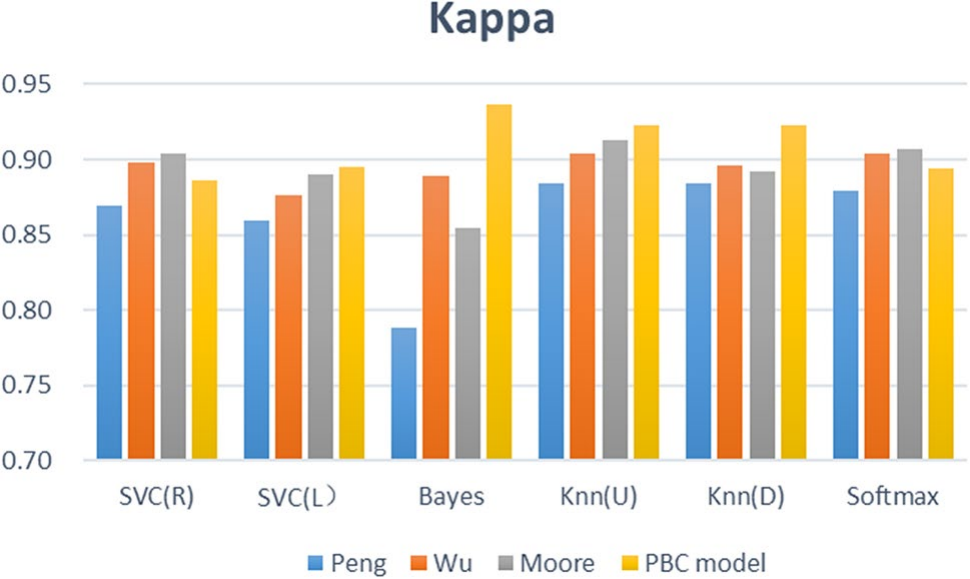
Kappa of four types of prediction models.

The feature fusion link is a critical step of the proposed framework, and the learning behaviour classification model directly determines the effect of feature fusion. We thus designed 3 comparative experiments (different learning behaviour classification models), built 4 types (24) of learning performance predictors based on 6 common machine learning algorithms, and analysed the proposed PBC model based on the prediction effects of these predictors. The effectiveness of the four types of learning performance predictor accuracy, F1-Score, and Kappa is shown in Figs. 9, 10, and 11.

Figure 9 describes the prediction accuracy of the four groups of experiments (PBC model group, Moore group, Wu group, Peng group). The accuracy of the PBC model group is between 95.44% and 97.40%; that of the Moore group is between 94.25% and 96.42%; that of the Wu group is between 95.01% and 96.10%; and that of the Peng group is between 90.89% and 95.34%. The PBC model group thus achieved a higher accuracy with the Naïve Bayes, KNN (U), and KNN (D) algorithms. Figure 10 shows the F1-score results of four sets of experiments. The F1-score of the six predictors based on the PBC model is between 0.9685 and 0.9818, that based on the Moore group is between 0.9606 and 0.9749, that based on the Wu group is between 0.9654 and 0.9727, and that based on the Peng group is between 0.9388 and 0.9677. The six algorithm models based on the PBC model all have higher F1-score results; the Moore-based and Wu-based F1-score performances are equivalent; and the Peng-based F1-score performance is the worst. Figure 11 shows the Kappa values of the comparative experiment. The Kappa interval based on the PBC model group is 0.8865 to 0.9364, that based on the Moore group is 0.8550 to 0.9126, that based on the Wu group is 0.8764 to 0.9043, and that based on the Peng group is 0.7881 0.8844. Thus, except for the SVC(R) algorithm, the Kappa of the other algorithms in the PBC model group are higher, and the indicators of the PBC model group are the most stable.

From Figs. 9, 10, and 11 , further analysis shows that the average accuracy rate of the PBC model group is 0.65%, 0.60%, and 2.02% higher than that of the Moore group, Wu group, and Peng group, respectively, and the upper (lower) limits of the accuracy increase by 0.98% (1.19%), 1.34% (0.33%), and 2.06% (4.56%); the average value of F1- score is higher by 0.45%, 0.41%, and 1.44%, and the upper (lower) limits of F1-score increase by 0.69% (0.79%), 0.90% (0.32%), and 1.41% (3.48%); the average values of Kappa are higher by 1.61%, 1.48%, and 4.86%, respectively, and the upper (lower) limits of Kappa increase by 2.38% (3.15%), 3.20% (1.00%), and 5.20% (9.84%). Thus, the predictor constructed based on the PBC model achieves the best accuracy, F1-score and Kappa value, and its prediction performance is better than that of the Moore classification method and Wu classification method, and markedly better than that of the Peng classification method. Therefore, when performing learning performance prediction tasks, it is effective and better to use the PBC model to divide learning behaviours.

## Conclusion

The learning performance predictor is an effective tool to ensure the quality of e-learning. How to build a learning performance predictor with high versatility and high accuracy has become a research hotspot in e-learning. This paper innovatively begins from the starting point of behaviour classification, introduces the learning behaviour feature fusion strategy to the traditional method, proposes the BCEP prediction framework, and proposes the PBC model based on a summary of existing e-learning behaviour classification methods. Experimental results with the OULAD dataset show that the BCEP prediction framework performs markedly better than the traditional learning performance predictor construction method, and the learning performance predictor constructed by this framework is accurate and stable. Subsequent experiments showed that the PBC model proposed in this paper as a feature fusion strategy of the prediction framework is effective and superior to other e-learning behaviour classification methods, and to a certain extent, also provides a new feasible scheme for quantitatively evaluating the pros and cons of e-learning behaviour classification methods.

In future work, we plan to build learning performance predictors for different e-learning platforms using the framework proposed in this article. By recording and analysing the performance of these predictors in real-world applications, we plan to optimize the proposed BCEP prediction framework. Concurrently, considering that the prediction targets for e-learning should be more diversified, in addition to the e-learning performance mentioned in this article, we plan to use similar methods to predict e-learning emotions to achieve better online supervision and early warning through multiangle prediction results and to ensure the quality of online learners’ learning.
